# Factors Associated With Unmet Supportive Care Needs and Emergency Department Visits and Hospitalizations in Ambulatory Oncology

**DOI:** 10.1001/jamanetworkopen.2023.19352

**Published:** 2023-06-21

**Authors:** Frank J. Penedo, Akina Natori, Sara E. Fleszar-Pavlovic, Vandana D. Sookdeo, Jessica MacIntyre, Heidy Medina, Patricia I. Moreno, Tracy E. Crane, Craig Moskowitz, Carmen L. Calfa, Matthew Schlumbrecht

**Affiliations:** 1 Department of Psychology, College of Arts and Science, University of Miami, Coral Gables, Florida; 2 Department of Medicine, Miller School of Medicine, University of Miami, Coral Gables, Florida; 3 Sylvester Comprehensive Cancer Center, University of Miami, Miami, Florida; 4 Division of Medical Oncology, Department of Medicine, Miller School of Medicine, University of Miami, Miami, Florida; 5 Department of Public Health Sciences, Miller School of Medicine, University of Miami, Miami, Florida; 6 Division of Hematology, Department of Medicine, Miller School of Medicine, University of Miami, Miami, Florida; 7 Division of Gynecologic Oncology, Department of Obstetrics and Gynecology, Miller School of Medicine, University of Miami, Miami, Florida

## Abstract

**Importance:**

Patients with cancer experience multiple supportive care needs (eg, coping and financial counseling) that, if not addressed, may result in poor clinical outcomes. Limited work has assessed the factors associated with unmet needs in large and diverse samples of ambulatory oncology patients.

**Objective:**

To characterize the factors associated with unmet supportive care needs among ambulatory oncology patients and to assess whether such needs were associated with emergency department (ED) visits and hospitalizations.

**Design, Setting, and Participants:**

Between October 1, 2019, and June 30, 2022, cross-sectional retrospective analyses were performed in a large and diverse ambulatory cancer population via My Wellness Check, an electronic health record (EHR)–based supportive care needs and patient-reported outcomes (PROs) screening and referral program.

**Main Outcomes and Measures:**

Demographic characteristics, clinical characteristics, and clinical outcomes were extracted from EHRs. Data on PROs (ie, anxiety, depression, fatigue, pain, and physical function), health-related quality of life (HRQOL), and supportive care needs were also collected. Logistic regressions examined factors associated with unmet needs. Cumulative incidence of ED visits and hospitalizations were assessed by Cox proportional hazards regression models adjusting for covariates.

**Results:**

The 5236 patients in the study had a mean (SD) age of 62.6 (13.1) years and included 2949 women (56.3%), 2506 Hispanic or Latino patients (47.9%), and 4618 White patients (88.2%); 1370 patients (26.2%) indicated Spanish as their preferred language, according to their EHR. A total of 940 patients (18.0%) reported 1 or more unmet needs. Black race (adjusted odds ratio [AOR], 1.97 [95% CI, 1.49-2.60]), Hispanic ethnicity (AOR, 1.31 [95% CI, 1.10-1.55]), 1 to 5 years after diagnosis (AOR, 0.64 [95% CI, 0.54-0.77]), more than 5 years after diagnosis (AOR, 0.60 [95% CI, 0.48-0.76]), anxiety (AOR, 2.25 [95% CI, 1.71-2.95]), depression (AOR, 2.07 [95% CI, 1.58-2.70]), poor physical function (AOR, 1.38 [95% CI, 1.07-1.79]), and low HRQOL scores (AOR, 1.89 [95% CI, 1.50-2.39]) were associated with greater unmet needs. Patients with unmet needs had a significantly higher risk of ED visits (adjusted hazard ratio [AHR], 1.45 [95% CI, 1.20-1.74]) and hospitalizations (AHR, 1.36 [95% CI, 1.13-1.63]) relative to patients without unmet needs.

**Conclusions and Relevance:**

In this cohort study of ambulatory oncology patients, unmet supportive care needs were associated with worse clinical outcomes. Patients from racial and ethnic minority groups and those with greater emotional or physical burden were more likely to have 1 or more unmet needs. Results suggest that addressing unmet supportive care needs may be crucial for improving clinical outcomes, and targeted efforts should focus on specific populations.

## Key Points

**Question:**

What factors are associated with patient-reported unmet supportive care needs in ambulatory oncology, and are these unmet needs associated with the risk of emergency department visits and hospitalizations?

**Findings:**

In this cohort study of 5236 ambulatory patients with cancer, Black race, Hispanic ethnicity, greater number of years after diagnosis, depression, poor physical function, and low health-related quality of life scores were associated with greater unmet needs. Compared with patients without unmet needs, those who reported having unmet needs had a significantly higher risk of emergency department visits and hospitalizations.

**Meaning:**

These findings suggest that unmet supportive care needs are associated with clinical outcomes, particularly in racial and ethnic minority populations; addressing these unmet needs is imperative for improving clinical outcomes, and efforts should target specific populations.

## Teaser

This cohort study characterizes the factors associated with unmet supportive care needs among ambulatory oncology patients and assesses whether such needs were associated with emergency department (ED) visits and hospitalizations.

## Introduction

In the past several decades, substantial advances in early detection and treatment options have improved overall survival rates for patients with cancer. Despite the benefits of survival, cancer treatments and their long-term adverse effects can be chronic and debilitating and can interfere with patients’ daily activities regardless of the disease stage or treatments received. Patients with cancer and survivors of cancer face unique physical and psychosocial needs that may interfere with patient care and adversely affect clinical outcomes.^1^ Cancer support services that address the many challenges faced by patients and survivors during the cancer experience (eg, emotional distress, practical needs such as financial concerns, and transportation) are essential to promote optimal care and well-being. Previous studies have identified various prevalent unmet supportive care needs, such as informational, physical, psychological, spiritual, and practical needs of daily living.^2,3^ A recent systematic review found that up to 79% of survivors of cancer may report at least 1 unmet need, with the financial, informational, psychological, and physical domains as the most commonly reported unmet supportive care needs.^4^ These unmet needs can vary across cancer diagnoses and along the care continuum. For example, long-term survivors of head and neck cancer (ie, mean, 5 years after diagnosis) needed more psychosocial and emotional support (eg, coping with death and dying), while patients immediately after treatment reported a greater need for informational support (eg, cancer diagnosis and progression information).^5^ These unmet supportive care needs can compromise adherence to treatment and negatively affect clinical outcomes if they are not adequately addressed. Conversely, addressing unmet needs by the provision of supportive care services, such as social work services, psychosocial support, and physical rehabilitation, may reduce adverse outcomes, including emergency department (ED) visits and hospitalizations. Prior work has documented a reduction in hospitalizations in ambulatory oncology clinics after supportive care services were provided to patients with cancer.^6^

Although previous research has identified unmet supportive care needs among patients with cancer, limited work has characterized patient-level factors that are associated with unmet needs. Several studies found that more severe anxiety and depression and lower health-related quality of life (HRQOL) among survivors of cancer were associated with greater reports of unmet supportive care needs.^2,5,7,8^ For example, patients with breast cancer who presented with higher levels of unmet needs immediately after treatment also reported more severe anxiety and depression.^5,9,10^ Unmet needs have also been associated with increased cancer-specific symptoms.^11^ These prior findings lack generalizability because most studies were conducted among small samples with limited representation regarding cancer site and stage, patients’ phases in the cancer care continuum, and race and ethnicity. Moreover, the association between unmet needs and clinical outcomes, specifically ED visits and hospitalizations as indicators of cumulative cancer burden and gaps in health care, has not been thoroughly examined, to our knowledge. Research is needed to examine the prevalence of unmet supportive care needs, the factors associated with unmet needs, and their association with clinical outcomes among a large and diverse ambulatory oncology population to effectively and systematically address the burden of the cancer experience at the patient and health system level.

Sylvester Comprehensive Cancer Center (SCCC) at the University of Miami Miller School of Medicine, Miami, Florida, implemented a routine electronic health record (EHR)–based patient-reported outcome (PRO) and needs screening system across the ambulatory oncology clinics. This retrospective cohort study assessed patient-reported unmet supportive care needs among a large and diverse ambulatory oncology population. We also evaluated whether unmet supportive care needs were associated with poor clinical outcomes, including ED visits and hospitalization.

## Methods

### Program Description

The My Wellness Check (MWC) assessment platform is designed to assess PROs and supportive care needs of patients with cancer and triage them to relevant services at the SCCC ambulatory oncology clinics. The program workflow is described in detail in prior publications.^12,13^ In brief, patients scheduled for an ambulatory oncology visit receive the MWC assessment via the patient portal. The questionnaire is scored and populated in the EHR with best practice alerts (BPAs) generated based on clinical cutoffs or stated supportive care needs. Key parameters for the questionnaire assignments, in addition to an upcoming appointment, include *International Statistical Classification of Diseases and Related Health Problems, Tenth Revision* cancer diagnosis, second or later visit to the ambulatory oncology clinic, and no prior MWC questionnaire completed in the past 30 days. Patients could answer the MWC questionnaire through the patient portal up to 72 hours before their next appointment. The MWC questionnaire was available in English and Spanish based on the patients’ selected preferred language in the EHR. Patients who completed the supportive care needs checklist in their first assigned MWC questionnaire between October 1, 2019, and June 30, 2022, were included in this study. The study protocol was approved by the institutional review board at the University of Miami. Informed consent from patients was waived because this is a retrospective medical record review study. This cohort study followed the Strengthening the Reporting of Observational Studies in Epidemiology (STROBE) reporting guideline.

### Symptom and Needs Assessment and BPAs

The MWC assessment consists of 5 Patient-Reported Outcomes Measurement Information System (PROMIS) computerized adaptive tests (CATs; anxiety, depression, pain interference, fatigue, and physical function) to assess emotional and physical symptoms commonly experienced by patients with cancer, as well as the Functional Assessment of Cancer Therapy–General (7-item version; FACT-G7) to assess HRQOL.^14,15,16,17,18,19,20,21^ In addition, supportive care needs are assessed by a 12-item checklist adapted from the National Comprehensive Cancer Network Distress Thermometer Problem Checklist (eg, financial concerns, transportation needs, and coping with cancer needs) and vetted by social workers, clinicians, and a patient and family advisory committee at SCCC.^22^ The details of each assessment are available in eTable 3 and eTable 4 in Supplement 1. The MWC questionnaires are not required and may be refused by patients.

PROMIS-CAT instruments are calibrated against a reference population and have a mean T score of 50 and a standard deviation of 10, with higher scores indicating poorer ratings in the health domain questions. The physical function PROMIS-CAT score is reversed and a lower T score means a worse physical score. Moderate or severe elevation in the PROMIS pain interference T score (≥70), fatigue T score (≥70), or physical function T score (≤30) trigger BPAs sent to the medical care team. Moderate or severe elevation in the PROMIS depression T score (≥60) or anxiety T score (≥65) and a report of any supportive care needs trigger automated BPAs sent to social work services. The social work team enters a disposition (eg, internal referral, external referral, or provided educational materials) in the EHR after they contact patients by telephone and discuss plans for the management of their needs. The medical care team clinicians address BPAs with a disposition during the clinical visit when they discuss the symptoms and needs with patients.

### Data Collection

#### Outcome Measures

The main outcomes were the time to an ED visit and hospitalization, which were calculated in days from the first MWC questionnaire assignment and the first event, respectively. All ED visits and hospitalizations in the University of Miami Health System were captured regardless of cause from the electronic data warehouse (EDW) that houses EHR data. No events outside of the network were captured.

#### Covariates

Self-reported patient demographic characteristics, as well as clinical characteristics such as cancer type or stage, treatment history, and Charlson Comorbidity Index (CCI), were collected from the EDW.^23^ Racial groups included Black, White, and other (American Indian or Alaska Native, Asian, Native Hawaiian or other Pacific Islander, and more than 1 race). Ethnic groups included Hispanic or Latino and non-Hispanic or Latino. Age and CCI were converted into binary variables (aged ≥65 years or <65 years; CCI ≤2 or >2). Patients were considered to be receiving active treatment when any cancer-directed treatments (ie, chemotherapy, radiotherapy, or immunotherapy) were initiated within 30 days from the MWC assessments. Health insurance status was categorized into 2 groups: insured (managed care, Medicare, or Medicaid) and uninsured (self-pay). Time since cancer diagnosis was calculated in years from the cancer diagnosis to the MWC assessment date and divided into 3 groups (<1 year, 1-5 years, and >5 years). Cancer stage was grouped into 3 categories: nonmetastatic (stage 0-III), metastatic (stage IV), and unknown. PROMIS-CAT T scores, FACT-G7 scores, reported supportive care needs, and related alerts with dispositions were also captured from the EDW. PROMIS-CAT T scores were converted to dichotomous variables using the thresholds mentioned. FACT-G7 scores were converted into a dichotomous variable with a cutoff value of 13 (≤13 indicates low HRQOL; >13 indicates high HRQOL).^24^ Best practice alert dispositions were not available for all patients in this study because they were implemented within the EHR 2 years after the MWC program was launched.

### Statistical Analysis

Descriptive statistics were calculated for demographic characteristics, clinical characteristics, and responses to the MWC questionnaire using χ^2^ tests. A stepwise logistic regression was used to examine variables associated with unmet supportive care needs with the prespecified level of significance for removal (*P* < .10) and for entry (*P* < .10). Adjusted odds ratios (AORs) and 95% CIs were obtained. The cumulative incidence function of ED visits and hospitalizations were estimated by the Kaplan-Meier method. Patients who were lost to follow-up or died were censored. The log-rank test was used to compare the outcomes between patients with and patients without unmet needs. Further analyses were performed using Cox proportional hazards regression models, adjusting for patient demographic characteristics, clinical characteristics, and PROs. Covariates were determined based on descriptive analyses and prior literature findings of factors associated with unmet supportive care needs.^2,5,7,8,11^ Exploratory analyses using Cox proportional hazards regression models were performed to compare clinical outcomes among patients without unmet needs, patients whose BPAs were completed, and patients whose BPAs were not completed. All *P* values were 2-sided, with *P* < .05 considered statistically significant. Data management and statistical analysis were performed with SAS, version 9.4 (SAS Institute Inc).

## Results

### Patient Demographic Characteristics

Between October 1, 2019, and June 30, 2022, 5236 of 12 563 patients completed their first assigned supportive care needs checklist in the MWC questionnaire. The 5236 patients had a mean (SD) age of 62.6 (13.1) years (2369 [45.2%] were aged 65 years or older) and included 2949 women (56.3%), 2506 Hispanic or Latino patients (47.9%), and 4618 White patients (88.2%); 1370 patients (26.2%) indicated Spanish as their preferred language, according to their EHR (Table 1). There were statistically significant differences in the patient demographic characteristics between patients with unmet needs and patients without unmet needs. The median time from diagnosis was 2.0 years (IQR, 0.6-4.5 years). A total of 3208 patients (61.3%) were receiving active treatments at the time of assessment.

**Table 1.** Demographic Characteristics of Patients

Characteristic	Patients, No. (%)		*P* value
	Without unmet needs (n = 4296)	With unmet needs (n = 940)	
Age, y			
<65	2293 (53.4)	574 (61.1)	<.001
≥65	2003 (46.6)	366 (38.9)	
Charlson Comorbidity Index			
≤2	1200 (27.9)	231 (24.6)	.04
>2	3096 (72.1)	709 (75.4)	
Sex			
Male	1920 (44.7)	367 (39.0)	.002
Female	2376 (55.3)	573 (61.0)	
Race			
Black	280 (6.5)	100 (10.6)	<.001
White	3813 (88.8)	805 (85.6)	
Other^a^	90 (2.1)	14 (1.5)	
Refused or not reported	113 (2.6)	21 (2.2)	
Ethnicity			
Hispanic or Latino	2002 (46.6)	504 (53.6)	<.001
Non-Hispanic or Latino	2139 (49.8)	402 (42.8)	
Refused or not reported	155 (3.6)	34 (3.6)	
Preferred language			
English	3216 (74.9)	650 (69.1)	<.001
Spanish	1080 (25.1)	290 (30.9)	
Health insurance			
Managed care	2341 (54.5)	587 (62.4)	<.001
Medicare	1257 (29.3)	196 (20.9)	
Medicaid	26 (0.6)	11 (1.2)	
Self-pay (no insurance)	72 (1.7)	15 (1.6)	
Cancer diagnosis			
Breast	813 (18.9)	168 (17.9)	.002
Hematology	658 (15.3)	130 (13.8)	
Digestive system	514 (12.0)	139 (14.8)	
Male genital system	485 (11.3)	86 (9.1)	
Head and neck	354 (8.2)	61 (6.5)	
Respiratory system	325 (7.6)	87 (9.3)	
Female genital system	329 (7.7)	97 (10.3)	
Other or unknown	818 (19.0)	172 (18.3)	
Cancer stage			
0	37 (0.9)	5 (0.5)	.15
I	348 (8.1)	77 (8.2)	
II	264 (6.1)	53 (5.6)	
III	213 (5.0)	63 (6.7)	
IV	217 (5.1)	58 (6.2)	
Unknown	3217 (74.9)	684 (72.8)	
Treatment status			
Active treatment	2540 (59.1)	668 (71.1)	<.001
No active treatment	1241 (28.9)	201 (21.4)	
Unknown	515 (12.0)	71 (7.6)	
Time since cancer diagnosis			
≤1 y	1362 (31.7)	412 (43.8)	<.001
1-5 y	1905 (44.3)	349 (37.1)	
>5 y	999 (23.3)	163 (17.3)	
Unknown	30 (0.7)	16 (1.7)	

^a^ Includes American Indian or Alaska Native, Asian, Native Hawaiian or other Pacific Islander, and more than 1 race.

### Questionnaire Responses and Dispositions of BPAs

A total of 940 patients (18.0%) reported 1 or more unmet supportive care needs within their first completed MWC questionnaire. Of these 940 patients, 651 patients had 1 unmet need, 165 patients had 2 unmet needs, and 124 patients had 3 or more unmet needs. Table 2 describes the reported unmet supportive care needs. Support for coping with a cancer diagnosis and financial concerns were the most reported unmet needs, followed by general cancer education and information. The completion rates of other assessments in the MWC questionnaire were as follows: PROMIS anxiety, 97.2% (n = 5088); PROMIS depression, 96.5% (n = 5053); PROMIS fatigue, 97.3% (n = 5094); PROMIS pain interference, 95.6% (n = 5008); PROMIS physical function, 96.5% (n = 5055); and FACT-G7; 93.4% (n = 4893). From these assessments, 418 patients (8.0%) had elevated PROMIS anxiety T scores, and 458 patients (8.7%) had elevated PROMIS depression T scores. A total of 701 patients (14.3%) met the clinical threshold for low HRQOL (ie, FACT-G7 scores ≤13). Table 2 presents additional PROMIS-CAT assessment results.

**Table 2.** Questionnaire Responses

Questionnaire response	Patients, No. (%)		*P* value
	Without unmet needs (n = 4296)	With unmet needs (n = 940)	
Supportive care needs checklist items^a^			
Support to help me cope with my diagnosis	NA	352 (37.4)	NA
Financial concerns	NA	323 (34.4)	NA
General cancer education and information	NA	231 (24.6)	NA
Advance directives	NA	157 (16.7)	NA
Transportation resources	NA	138 (14.7)	NA
Sexual health concerns	NA	59 (6.3)	NA
Housing needs	NA	51 (5.4)	NA
Work or school concerns	NA	42 (4.7)	NA
Family problems or family health concerns	NA	25 (2.7)	NA
Oncofertility	NA	20 (2.1)	NA
Spiritual concerns	NA	18 (1.9)	NA
Childcare	NA	6 (0.6)	NA
PROMIS-CAT, No./total No. (%)^b^			
Anxiety (T score ≥65)	215/4167 (5.2)	203/921 (22.0)	<.001
Depression (T score ≥60)	241/4136 (5.8)	217/917 (23.7)	<.001
Fatigue (T score ≥70)	88/4208 (2.1)	78/840 (9.3)	<.001
Pain interference (T score ≥70)	121/4096 (3.0)	88/912 (9.6)	<.001
Poor physical function (T score ≤30)	308/4144 (7.4)	171/911 (18.8)	<.001
Low FACT-G7 score (≤13)	419/4012 (10.4)	282/872 (32.3)	<.001

Abbreviations: FACT-G7, Functional Assessment of Cancer Therapy-General 7-item version; NA, not applicable; PROMIS-CAT, Patient-Reported Outcomes Measurement Information System computerized adaptive test.

^a^ One patient can report multiple needs in 1 assessment.

^b^ Denominators vary because of incomplete assessments.

During the study period, 940 BPAs were triggered by the supportive care needs checklists. However, 526 BPA dispositions (56.0%) were missing because they were addressed before dispositions were coded in an extractable format (ie, before August 2021). Another 115 BPA dispositions (12.2%) were missing between May and June 2022 during the system upgrade. At the time of data cutoff, 76 BPAs were still not addressed. The rest of 223 BPAs were addressed with the following dispositions: provided general education (n = 81 [8.6%]), discussed with a patient (n = 53 [5.6%]), internal referral (n = 26 [2.8%]), external referral (n = 15 [1.6%]), both internal and external referrals (n = 2 [0.2%]), and unable to contact a patient after 3 attempts (n = 46 [4.9%]).

### Factors Associated With Unmet Supportive Care Needs

Table 3 shows the univariate and multivariate logistic regression analyses assessing the factors associated with unmet supportive care needs. Multivariate logistic regression analysis revealed that Black race (AOR, 1.97 [95% CI, 1.49-2.60]), Hispanic ethnicity (AOR, 1.31 [95% CI, 1.10-1.55]), 1 to 5 years after diagnosis (AOR, 0.64 [95% CI, 0.54-0.77]), more than 5 years after diagnosis (AOR, 0.60 [95% CI, 0.48-0.76]), anxiety (AOR, 2.25 [95% CI, 1.71-2.95]), depression (AOR, 2.07 [95% CI, 1.58-2.70]), poor physical function (AOR, 1.38 [95% CI, 1.07-1.79]), and low HRQOL as indicated by low FACT-G7 scores (AOR, 1.89 [95% CI, 1.50-2.39]) were associated with having unmet supportive care needs.

**Table 3.** Factors Associated With Unmet Supportive Care Needs

Variable	Univariate		Multivariate	
	OR (95% CI)	*P* value	OR (95% CI)	*P* value
Demographic characteristics				
Aged ≥65 y (reference: aged <65 y)	0.73 (0.63-0.84)	<.001	0.86 (0.73-1.02)	.08
Male (reference: female)	0.80 (0.69-0.72)	.002	NA	NA
Race (reference: White)				
Black	1.69 (1.33-2.15)	<.001	1.97 (1.49-2.60)	<.001
Other^a^	0.75 (0.48-1.17)	.21	0.88 (0.47-1.62)	.67
Hispanic (reference: non-Hispanic)	1.34 (1.16-1.55)	<.001	1.31 (1.10-1.55)	.003
Spanish speaker (reference: English speaker)	1.33 (1.14-1.55)	<.001	NA	NA
Uninsured (reference: insured)	0.95 (0.54-1.67)	.86	NA	NA
Clinical characteristics				
High CCI (reference: CCI low)	1.19 (1.01-1.40)	.04	NA	NA
Active treatment (reference: no active treatment)	1.62 (1.37-1.93)	<.001	1.21 (0.99-1.46)	.05
Metastatic cancer (reference: nonmetastatic cancer)	1.16 (0.84-1.61)	.37	NA	NA
Time since cancer diagnosis (reference: <1 y)				
1-5 y	0.60 (0.52-0.71)	.02	0.64 (0.54-0.77)	<.001
>5 y	0.54 (0.44-0.66)	<.001	0.60 (0.48-0.76)	<.001
Cancer type (reference: breast cancer)				
Digestive system	1.31 (1.12-1.68)	.04	NA	NA
Female genital system	1.43 (1.08-1.89)	.01	NA	NA
Head and neck	0.83 (0.61-1.15)	.26	NA	NA
Hematology	0.96 (0.74-1.23)	.73	NA	NA
Respiratory system	1.30 (0.97-1.73)	.07	NA	NA
Male genital system	0.86 (0.65-1.14)	.29	NA	NA
Other or unknown	1.02 (0.81-1.29)	.89	NA	NA
Patient-reported outcomes				
Anxiety	5.20 (4.22-6.40)	<.001	2.25 (1.71-2.95)	<.001
Depression	5.01 (4.10-6.12)	<.001	2.07 (1.58-2.70)	<.001
Pain	3.51 (2.64-4.66)	<.001	NA	NA
Fatigue	4.63 (3.38-6.35)	<.001	1.43 (0.97-2.13)	.07
Poor physical function	2.88 (2.35-3.53)	<.001	1.38 (1.07-1.79)	.01
Low FACT-G7 score (≤13)	4.11 (3.45-4.89)	<.001	1.89 (1.50-2.39)	<.001

Abbreviations: CCI, Charlson Comorbidity Index; FACT-G7, Functional Assessment of Cancer Therapy–General (7-item version); OR, odds ratio; NA, not applicable.

^a^ Includes American Indian or Alaska Native, Asian, Native Hawaiian or other Pacific Islander, and more than 1 race.

### Unmet Supportive Care Needs and Clinical Outcomes

**Figure 1.**
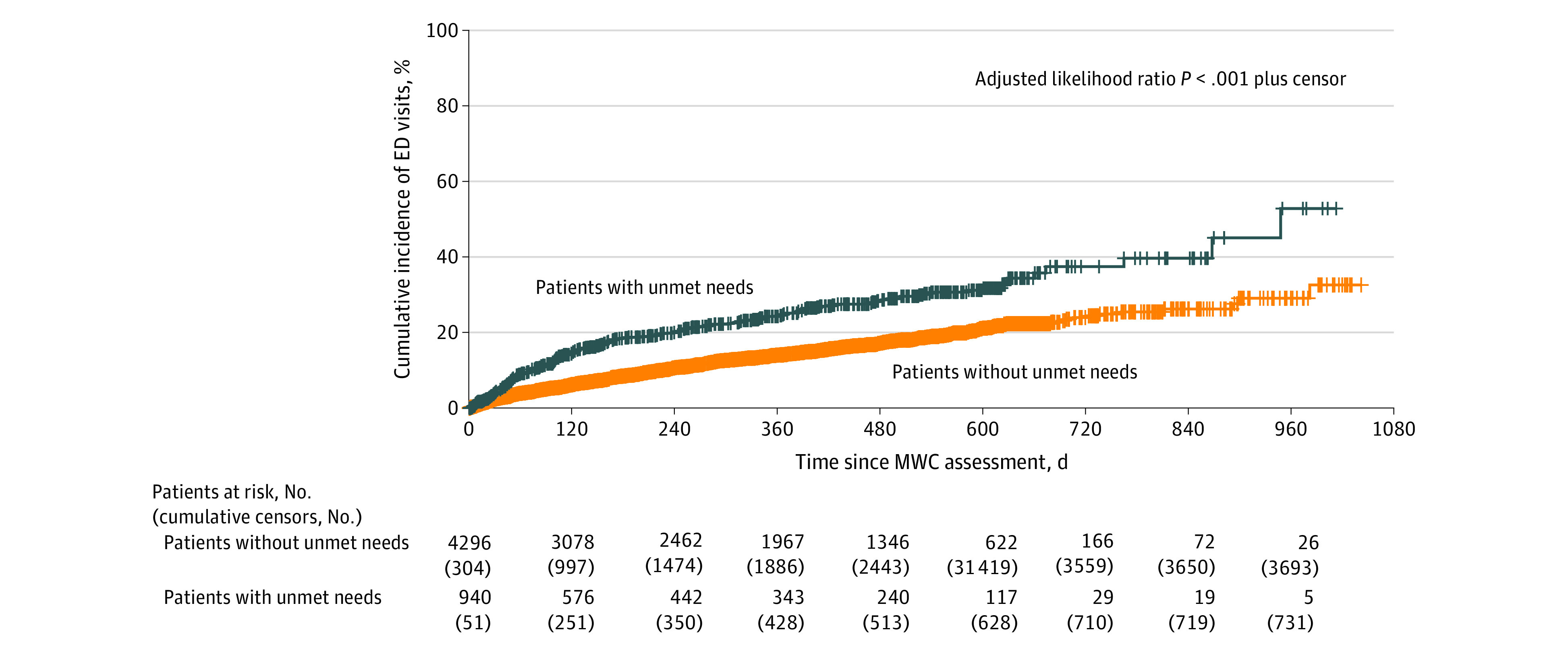
Cumulative Incidence of Emergency Department (ED) Visits Among Patients With Unmet Supportive Care Needs vs Patients Without Unmet Needs There were a total of 578 events among 4296 patients without unmet needs and 120 events among 940 patients with unmet needs (hazard ratio, 1.45; 95% CI, 1.20-1.74). MWC indicates My Wellness Check.

**Figure 2.**
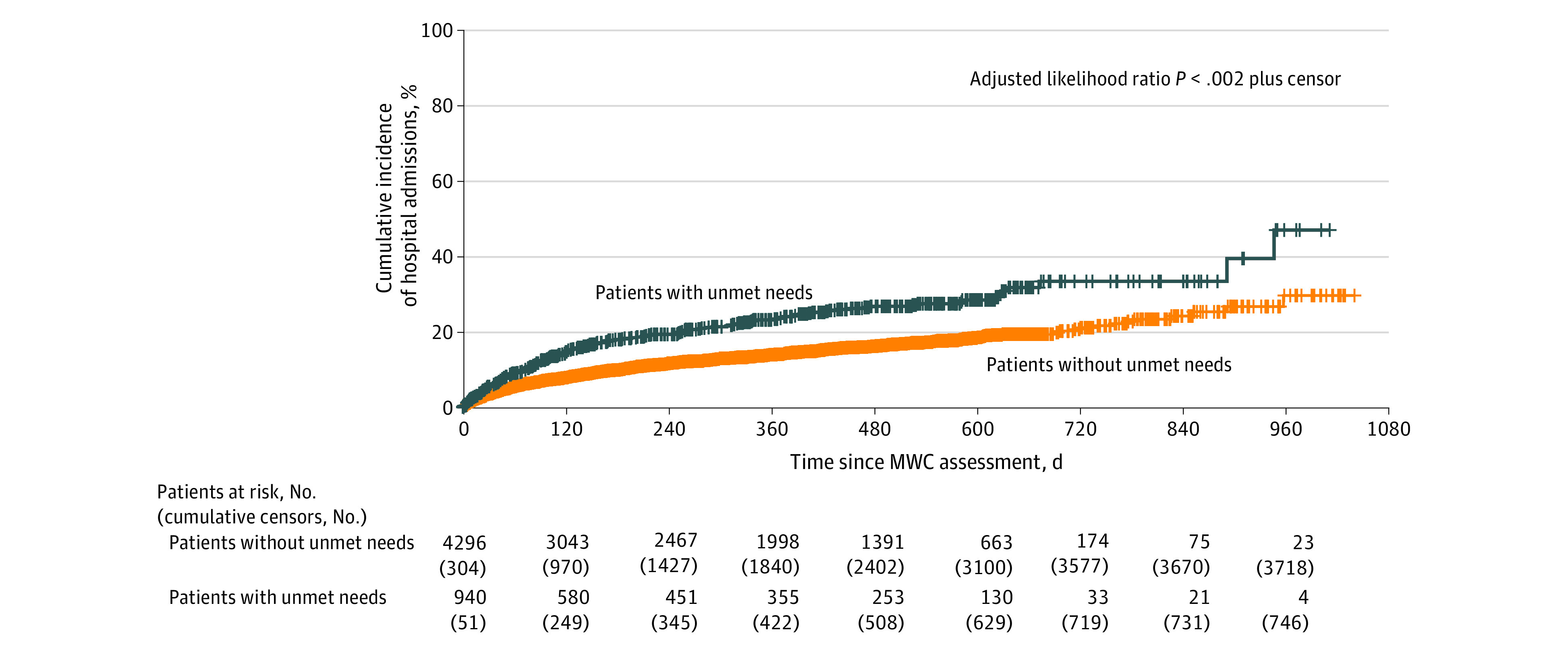
Cumulative Incidence of Hospital Admissions Among Patients With Unmet Supportive Care Needs vs Patients Without Unmet Needs There were a total of 555 events among 4296 patients without unmet needs and 190 events among 940 patients with unmet needs (hazard ratio, 1.36; 95% CI, 1.13-1.63). MWC indicates My Wellness Check.

Greater risks of ED visits and hospitalizations were observed among patients with unmet supportive care needs compared with those without unmet needs (ED visits, 24.7% vs 14.2% at 360 days; *P* < .001 [Figure 1]; and hospitalizations, 23.3% vs 13.9% at 360 days; *P* < .001 [Figure 2]). Patients with unmet needs were at a significantly higher risk of ED visits (adjusted hazard ratio [AHR], 1.45 [95% CI, 1.20-1.74]) and higher risk of hospitalizations (AHR, 1.36 [95% CI, 1.13-1.63]) after adjusting for covariates (eTable 1 in Supplement 1). In the exploratory analysis (eTable 2 in Supplement 1), the risks of ED visits and hospitalization of patients with incomplete BPAs were not significantly different compared with patients with completed BPAs (ED visits: AHR, 1.84 [95% CI, 0.97-3.50]; hospitalizations: AHR, 1.87 [95% CI, 0.98-3.56] for hospitalizations) (eFigures 1 and 2 in Supplement 1).

## Discussion

The present study is, to our knowledge, the most comprehensive assessment of unmet supportive care needs among ambulatory oncology patients. The study includes a diverse population, incorporating a high proportion of Hispanic patients (47.9%), various primary cancer sites, and patients across multiple phases of the cancer care continuum. We found that 18.0% of patients reported 1 or more unmet supportive care needs. A systematic review reported that the prevalence of unmet needs was highly variable (range, 1%-93%) within and between studies given the different assessments or questionnaires and study populations (cancer type and phase of the cancer journey).^3^ It also highlighted that the highest level of unmet needs was observed among patients undergoing active treatment (ie, chemotherapy, radiotherapy, or immunotherapy). The observed prevalence of unmet needs was relatively low, although 61.3% of our study population were receiving active treatments at the time of assessment. One of the possible explanations for this difference could be that our study patients were relatively “experienced” patients (median time from cancer diagnosis, 2.0 years) who had established relationships with cancer support services before the assessment. Another possible reason is the difference in the health care structure from other studies. Studies reporting a high prevalence of unmet needs were conducted in Canada, the UK, and Australia, where universal public health insurance programs are available.^7,25,26^ Although health insurance status was not associated with unmet needs in this study, the health care structure and variation in health care funding models may have some implications for the extent of unmet needs.

We found that higher anxiety, higher depression, decreased physical function, and low HRQOL scores were the factors associated with unmet supportive care needs. This finding is consistent with several cross-sectional studies.^2,5,7,8,9,10^ Given the cross-sectional design of the present study and other studies, further research with longitudinal designs are needed to determine whether the changes in anxiety, depression, and HRQOL are associated with changes in unmet needs, or vice versa, or whether they have a bidirectional association. We also found that Black race and Hispanic ethnicity were associated with greater unmet needs. Identifying as Black race or Hispanic ethnicity is not a risk factor for greater unmet needs; however, social constructs create and perpetuate systemic racism and discrimination, which are associated with disparate health outcomes and limited resources for racial and ethnic minority populations.^27^ Study findings align with the well-evidenced disparities in cancer care delivery by race and ethnicity due to structural, socioeconomic, socioenvironmental, and behavioral factors.^28,29^ These findings suggest that cancer support services with a risk stratification strategy based on demographic and clinical factors to prioritize these vulnerable populations are needed.

The present study showed a significant difference in clinical outcomes between patients with unmet needs and those who did not report unmet needs. Patients with unmet needs had a 45% higher risk of ED visits and a 36% higher risk of hospitalization than patients without unmet needs, even after adjusting for demographic and clinical characteristics. This exploratory analysis did not show significant differences in the risks of ED visits and hospitalization between patients with incomplete BPAs and patients with completed BPAs. The lack of statistical significance was likely due to insufficient power (the number of patients with incomplete BPAs was 76, with 10 ED visits and 12 hospitalizations), rather than due to a true absence of difference. Previous studies noted that unmet supportive care needs were associated with less frequent patient-clinician communication and less satisfaction with cancer care, which are established, critical factors for adherence to cancer treatment.^11,30,31^ Unlike the other factors that we identified as independent risk factors for an ED visit and hospitalization (age, sex, race, ethnicity, and CCI), patient-reported unmet needs are modifiable. Thus, reducing unmet needs may be a critical target for interventions to improve cancer treatment adherence and clinical outcomes.

### Limitations

Although this study significantly contributed to our understanding of the factors associated with unmet needs and the association between unmet needs and clinical outcomes, several limitations should be considered. First, despite prospectively examining the association of unmet supportive care needs with ED visits and hospitalizations, supportive care needs were assessed only at the first MWC administration. A longitudinal study that examines the change in unmet needs over time is warranted. Second, the reason for an ED visit and hospitalization could not be accurately procured retrospectively. Thus, preplanned hospitalizations and nononcology-related events were included in our outcomes. Third, this study was conducted in a large university-based, National Cancer Institute–designated cancer center with robust survivorship and supportive care resources, and results may not be generalizable to less-resourced settings. Fourth, BPA dispositions were not adequately captured in the EDW during the study period, and the effectiveness of addressing BPAs was not able to be fully analyzed in this study. The study team is currently collecting BPA dispositions, and future analyses will examine how BPA dispositions are associated with clinical outcomes. Fifth, sensitivity analyses were not conducted; thus, the study’s conclusions may be overly reliant on a single set of assumptions and parameter values, which could limit the generalizability of the findings. Moreover, without sensitivity analyses, it is difficult to evaluate the potential association of unmeasured confounding variables or other sources of bias with clinical outcomes. Therefore, the lack of sensitivity analyses should be taken into consideration when interpreting the findings and may warrant further investigation in future study analyses.

## Conclusion

In this cohort study of ambulatory oncology patients, unmet supportive care needs were associated with unfavorable clinical outcomes, including a higher risk for ED visit and hospitalization. Patients with cancer who are from racial or ethnic minority groups and those with more significant emotional or physical burdens were more likely to have 1 or more unmet needs. These findings suggest that addressing unmet supportive care needs is imperative for improving clinical outcomes and that efforts to address unmet needs should target specific populations.

## Notes

Supplement 1. **eTable 1.** Factors Associated With Emergency Room Visits and Hospital Admissions **eTable 2.** Clinical Outcomes Associations With Incomplete BPAs, Completed BPAs, and No Unmet Needs Endorsed **eTable 3.** Details of PROMIS-CATs **eTable 4.** List of Options in the Supportive Care Needs Assessment **eFigure 1.** Cumulative Incidence of ER Visits Among Patients With Incomplete BPAs, Patients With Completed BPAs, and Patients With No Unmet Needs Endorsed **eFigure 2.** Cumulative Incidence of Hospital Admissions Among Patients With Incomplete BPAs, Patients With Completed BPAs, and Patients With No Unmet Needs Endorsed

Supplement 2.
Data Sharing Statement
