# Supplementary material for: Factors Associated With Unmet Supportive Care Needs and Emergency Department Visits and Hospitalizations in Ambulatory Oncology
Source: JAMA Netw Open. 2023 Jun 21;6(6):e2319352. doi: 10.1001/jamanetworkopen.2023.19352 (PMC10285575; doi:10.1001/jamanetworkopen.2023.19352)
Supplement: Supplement 1. — eTable 1. Factors Associated With Emergency Room Visits and Hospital Admissions eTable 2. Clinical Outcomes Associations With Incomplete BPAs, Completed BPAs, and No Unmet Needs Endorsed eTable 3. Details of PROMIS-CATs eTable 4. List of Options in the Supportive Care Needs Assessment eFigure 1. Cumulative Incidence of ER Visits Among Patients With Incomplete BPAs, Patients With Completed BPAs, and Patients With No Unmet Needs Endorsed eFigure 2. Cumulative Incidence of Hospital Admissions Among Patients With Incomplete BPAs, Patients With Completed BPAs, and Patients With No Unmet Needs Endorsed [file jamanetwopen-e2319352-s001.pdf]

Supplementary Online Content

Penedo FJ, Natori A, Fleszar-Pavlovic SE, et al. Factors associated with unmet supportive care needs and emergency department visits and hospitalizations in ambulatory oncology. *JAMA Netw Open.* 2023;6(6):e2319352. doi:10.1001/jamanetworkopen.2023.19352

eTable 1. Factors Associated With Emergency Room Visits and Hospital Admissions
eTable 2. Clinical Outcomes Associations With Incomplete BPAs, Completed BPAs, and No Unmet Needs Endorsed
eTable 3. Details of PROMIS-CATs
eTable 4. List of Options in the Supportive Care Needs Assessment
eFigure 1. Cumulative Incidence of ER Visits Among Patients With Incomplete BPAs, Patients With Completed BPAs, and Patients With No Unmet Needs Endorsed
eFigure 2. Cumulative Incidence of Hospital Admissions Among Patients With Incomplete BPAs, Patients With Completed BPAs, and Patients With No Unmet Needs Endorsed

This supplementary material has been provided by the authors to give readers additional information about their work.

© 2023 Penedo FJ et al. *JAMA Network Open.*

eTable 1. Factors Associated With Emergency Room Visits and Hospital Admissions

ER visit	Univariate		Multivariate	
Predictor	HR (95%CI)	P value	HR (95%CI)	P value
Age	0.99 (0.98-0.99)	0.004	0.99 (0.98-0.99)	0.002
Male (vs. Female)	0.97 (0.94-1.12)	0.66	NA	NA
Race				
White (ref)	-	-	-	-
Black	1.43 (1.13-1.82)	0.003	1.60 (1.22-2.09)	<.001
Other	0.99 (0.67-1.48)	0.98	1.20 (0.79-1.82)	0.40
Hispanic (vs. Non-Hispanic)	0.45 (1.25-1.68)	<.001	1.39 (1.17-1.66)	<.001
Charlson Score	1.17 (1.15-1.20)	<.001	1.16 (1.13-1.18)	<.001
Uninsured (vs. insured)	1.65 (1.02-2.66)	0.04	1.16 (0.64-2.11)	0.62
Active treatment	2.64 (2.15-3.23)	<.001	2.08 (1.67-2.59)	<.001
Patient-reported outcomes				
Anxiety	2.19 (1.80-2.66)	<.001	1.49 (1.14-1.96)	0.004
Depression	1.98 (1.63-2.41)	<.001	0.94 (0.71-1.25)	0.67
Pain	2.78 (2.15-3.60)	<.001	1.39 (0.98-1.98)	0.07
Fatigue	2.09 (1.50-2.91)	<.001	0.92 (0.62-1.38)	0.71
Declined physical function	2.55 (2.11-3.09)	<.001	1.54 (1.20-1.99)	<.001
Unmet supportive care needs	1.85 (1.58-2.17)	<.001	1.45 (1.20-1.74)	<.001
Hospitalization	Univariate		Multivariate	
Predictor	HR (95%CI)	P value	HR (95%CI)	P value
Age	0.99 (0.98-0.99)	<.001	0.98 (0.97-0.99)	<.001
Male (vs. Female)	1.20 (1.04-1.39)	0.01	1.53 (1.15-1.80)	<.001
Race				
White (ref)	-	-		
Black	1.35 (1.06-1.72)	0.01	1.63 (1.24-2.13)	<.001
Other	0.98 (0.66-1.46)	0.92	1.39 (0.94-2.07)	0.10
Ethnicity				
Non-Hispanic (ref)	-	-		
Hispanic	1.17 (1.01-1.36)	0.03	1.23 (1.03-1.47)	0.02
Charlson Score	1.20 (1.18-1.22)	<.001	1.16 (1.14-1.19)	<.001
Uninsured (vs. insured)	1.57 (0.96-2.58)	0.07	1.25 (0.72-2.18)	0.42
Active treatment	7.25 (5.34-9.83)	<.001	5.88 (4.25-8.11)	<.001
Patient-reported outcomes				
Anxiety	1.67 (1.35-2.07)	<.001	0.96 (0.71-1.29)	0.78
Depression	1.80 (1.46-2.01)	<.001	0.99 (0.73-1.33)	0.94
Pain	2.35 (1.79-3.08)	<.001	1.14 (0.79-1.65)	0.48
Fatigue	2.10 (1.51-2.92)	<.001	1.03 (0.70-1.54)	0.87
Declined physical function	2.50 (2.06-3.03)	<.001	1.59 (1.24-2.05)	<.001
Unmet supportive care needs	1.73 (1.47-2.05)	<.001	1.36 (1.13-1.63)	0.001

CI; confidence interval, HR; hazard ratio, ref: reference

© 2023 Penedo FJ et al. *JAMA Network Open.*

eTable 2. Clinical Outcomes Associations With Incomplete BPAs, Completed BPAs, and No Unmet Needs Endorsed

Predictor	ER visit		Hospitalization	
	aHR (95%CI)	P value	aHR (95%CI)	P value
Age	0.99 (0.98-0.10)	0.001	0.98 (0.97-0.99)	<.001
Male (vs. Female)	1.13 (0.95 -1.33)	0.16	1.52 (1.28-1.80)	<.001
Race				
White (ref)	-	-	-	-
Black	1.60 (1.22-2.10)	<.001	1.61 (1.23-2.12)	<.001
Other	1.21 (0.79-1.83)	0.38	1.41 (0.95-2.09)	0.09
Ethnicity				
Non-Hispanic (ref)	-	-	-	-
Hispanic	1.41 (1.19-1.69)	<.001	1.22 (1.02-1.46)	0.03
Charlson Score	1.15 (1.13-1.18)	<.001	1.17 (1.14-1.19)	<.001
Uninsured (vs. insured)	1.21 (0.66-2.20)	0.54	1.31 (0.75-2.28)	0.34
Active treatment	2.12 (1.70-2.64)	<.001	5.93 (4.28-8.23)	<.001
Patient-reported outcomes				
Anxiety	1.50 (1.14-1.97)	0.004	0.97 (0.72-1.31)	0.86
Depression	0.96 (0.72-1.29)	0.79	0.99 (0.73-1.35)	0.96
Pain	1.36 (0.95-1.95)	0.09	1.13 (0.78-1.6)	0.52
Fatigue	0.93 (0.63-1.39)	0.93	1.04 (0.69-1.55)	0.85
Declined physical function	1.56 (1.21-2.02)	<.001	1.62 (1.25-2.09)	<.001
Unmet SCN BPA status				
Completed BPA (ref)	-	-	-	-
Incomplete BPA	1.84 (0.97-3.50)	0.06	1.87 (0.98-3.56)	0.06
No SCN endorsed	0.71 (0.59-0.86)	<.001	0.78 (0.64-0.95)	0.02

aHR: adjusted hazard ratio, ref: reference, SCN: supportive care needs

© 2023 Penedo FJ et al. *JAMA Network Open.*

eTable 3. Details of PROMIS-CATs

Domain	Item Bank	Number of items in the item bank	Number of items to be administered*
Anxiety	PROMIS Bank v1.0	29	4-12
Depression	PROMIS Bank v1.0	28	4-12
Pain Interference	PROMIS Bank v1.1	95	4-12
Fatigue	PROMIS Bank v1.0	40	4-12
Physical Function	PROMIS Bank v2.0 (English)<br>PROMIS Bank v1.2 (Spanish)	165	4-12

*PROMIS measures are administered by a computerized adaptive test (CAT). CAT is a type of measure in which questions a person answers are tailored to that person by item response theory. CATs begin from an item bank which is a collection of items (questions) all measuring the same function/symptom and these items are ordered by level of difficulty/severity from low to high. CATs administer the first item which is one in the middle of the range of function/symptom severity. After the first response, the CAT algorithm using IRT selects the best item in the item bank based on an estimated score obtained by the prior response. The items are assigned until a standard error of 2 or less has been reached or 12 items were answered.

© 2023 Penedo FJ et al. *JAMA Network Open.*

eTable 4. List of Options in the Supportive Care Needs Assessment

Prompt "I would like for a treatment team member to contact me for help with (check all that apply):"
No needs at this time
Support to help me cope with my illness and/or manage stress
Financial/insurance concerns about my health care
Transportation resources
General education and information
Advance directives: medical actions to be taken if my health prevents me from making decisions (living will, power of attorney, health care surrogate)
Family problems/family health concerns
Sexual health concerns
Housing needs/concerns
Oncofertility (ability to have children)
Work/school concerns
Spiritual/religious concerns
Childcare

© 2023 Penedo FJ et al. *JAMA Network Open.*

eFigure 1. Cumulative Incidence of ER Visits Among Patients With Incomplete BPAs, Patients With Completed BPAs, and Patients With No Unmet Needs Endorsed

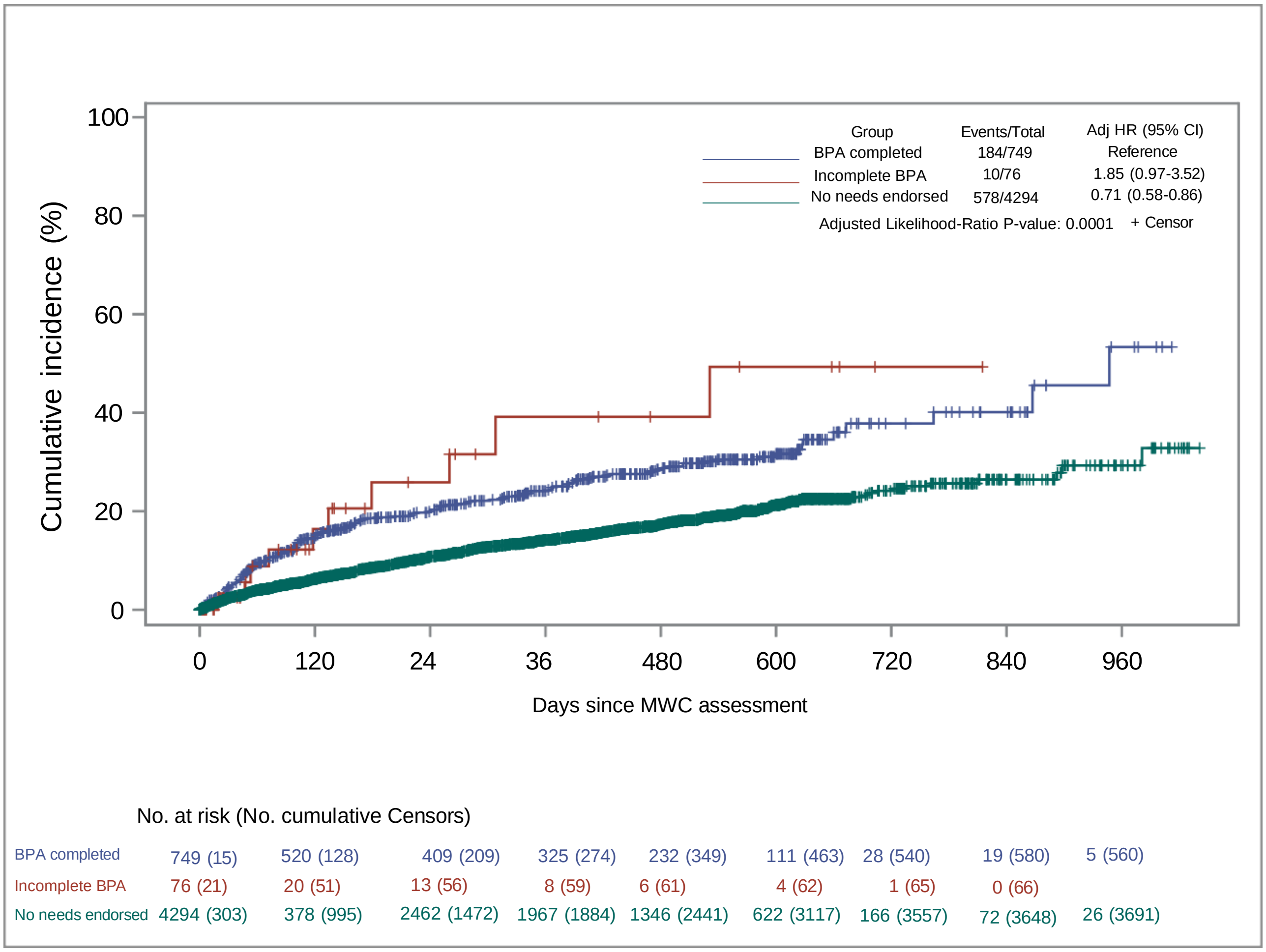

© 2023 Penedo FJ et al. *JAMA Network Open.*

eFigure 2. Cumulative Incidence of Hospital Admissions Among Patients With Incomplete BPAs, Patients With Completed BPAs, and Patients With No Unmet Needs Endorsed

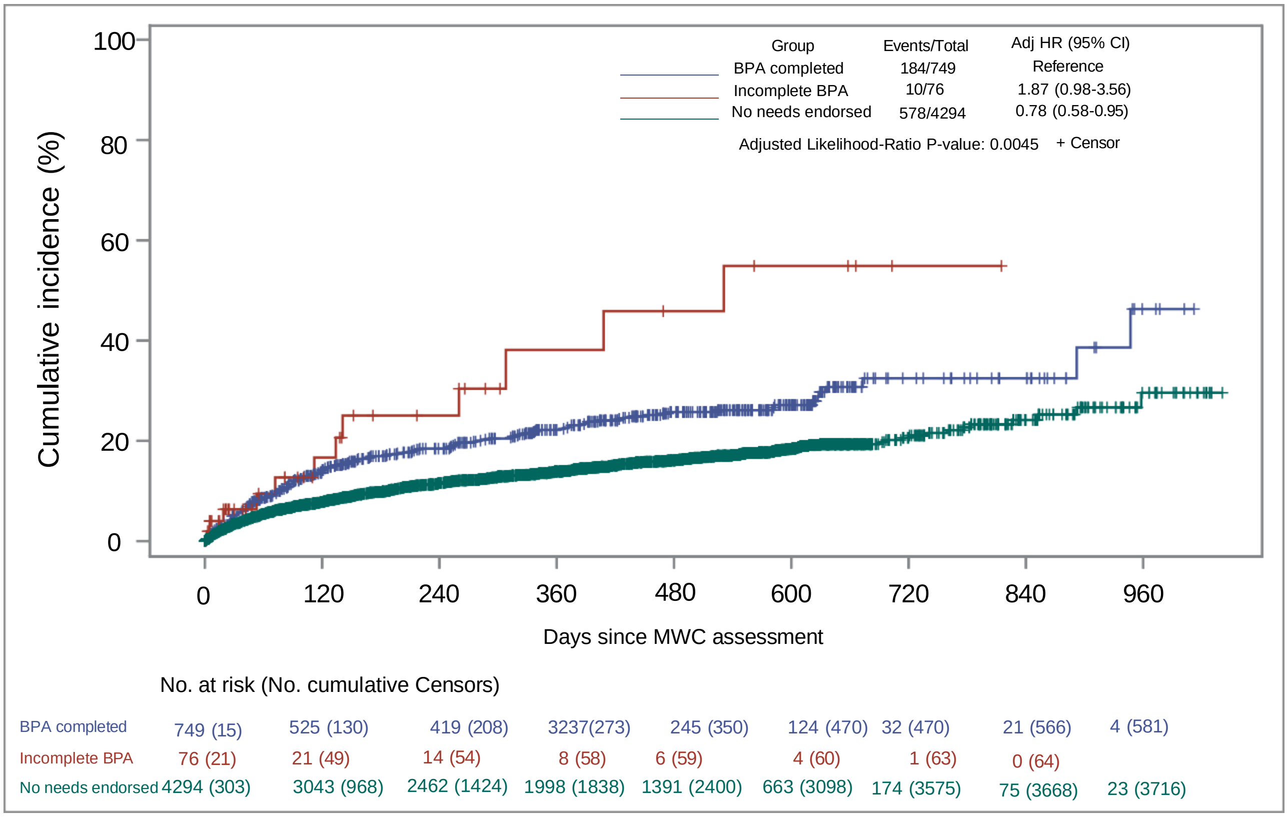

© 2023 Penedo FJ et al. *JAMA Network Open.*
