# Supplementary material for: Factors Associated With Unmet Supportive Care Needs and Emergency Department Visits and Hospitalizations in Ambulatory Oncology
Source: JAMA Netw Open. 2023 Jun 21;6(6):e2319352. doi: 10.1001/jamanetworkopen.2023.19352 (PMC10285575; doi:10.1001/jamanetworkopen.2023.19352)
Supplement: Supplement 2. — Data Sharing Statement [file jamanetwopen-e2319352-s002.pdf]

Data Sharing Statement

Penedo. Factors Associated With Unmet Supportive Care Needs and Emergency Department Visits and Hospitalizations in Ambulatory Oncology. *JAMA Netw Open*. Published June 21, 2023. doi:10.1001/jamanetworkopen.2023.19352

Data
Data available: No
